# The Wastes of Sanitary Ceramics as Recycling Aggregate to Special Concretes

**DOI:** 10.3390/ma11081275

**Published:** 2018-07-24

**Authors:** Paweł Ogrodnik, Jacek Szulej, Wojciech Franus

**Affiliations:** 1 The Main School of Fire Service, Faculty of Fire Safety Engineering, 52/54 Słowackiego Str., 01-629 Warszawa, Poland; pogrodnik@sgsp.edu.pl; 2 Lublin University of Technology, Faculty of Civil Engineering and Architecture, 40 Nadbystrzycka Str., 20-618 Lublin, Poland; w.franus@pollub.pl

**Keywords:** recycling, sanitary ceramics, concrete, recycling aggregate

## Abstract

This article presents the results of research on the wastes of sanitary ceramics as an aggregate to concretes. The case of high temperature load was taken into account. Six concrete mixes were designed on Portland and calcium aluminate cement with various content of aerating admixture. Only the ground waste ceramics were used as an aggregate from one of the Polish sanitary ceramics plants. The abrasion test by Boehme blade of the designed concrete was conducted within the frame of study and compression strength tests on the cylindrical samples were performed as well. Some samples were initially annealed at 400 or 800 °C prior to strength tests. In order to determine the impact of annealing on the phase content and the concrete sample structure, the analyses on phase content (XRD—X-ray diffraction) and scanning electron microscopy (SEM) were conducted. The tests on compression strength demonstrated that there is considerable resistance of concrete containing ceramic aggregate and calcium aluminate cement to high temperatures. Abrasion tests confirmed that selected mixes have a high resistance to abrasion and they can be applied as a concrete coating. The possibility of ceramic cullet use as an aggregate to special concretes has been confirmed by the conducted research on specific features. Taking into consideration the available literature, the article presents widely conducted research in the area of the internal structure of concrete designed on the basis of recycled ceramic aggregate, the phase content of individual components, and basic mechanical tests both in normal temperatures and under thermal stress.

## 1. Introduction

The growth of concrete use in the world simultaneously generates increased demand on resources that are essential for its production. At the same time, the growth of social awareness as well as concerns related to environmental protection reflected in the new legislation incline the concrete industry to decrease the emission of greenhouse gases and the use of natural resources as well [1,2]. This action falls within the concept of sustainable development for which it is extremely important to cut down on the energy consumption and the use of natural resources according to the rule 4R (Reduce-Reuse-Recover-Recycle) [3]. Due to the decrease of natural resources in specific areas, many resources must be transported over long distances. As a result, their prices are raised, simultaneously causing greater interest in aggregates to concrete obtained from recycling and by-products of production in the form of admixtures [4,5,6]. Currently, brick, glass, tire and recycled concrete aggregates (RCA) are frequently applied as a recycled aggregate. According to data presented by [7], the most common recycled aggregate in the USA is a concrete aggregate (RCA) whose share in the market constitutes 54%. Its cost is relatively low, and the likelihood of obtaining it is substantially due to the fact that there is a necessity to demolish buildings and engineering facilities. The use of fly dust constitutes 20% of the recycled aggregate market. The consumption of remaining waste materials as aggregates is as following: slag 11%, microsilica 9%, and rubber tires 4%. The issue of optimal use of concrete rubble obtained from concrete structure demolition has lately been the subject of research and decisive actions in highly industrialized countries.

The key parameter of recycled concrete aggregates is their density which as a rule is lower in comparison to the density of natural aggregate. It also has an impact on considerable water absorption, lower strength, and on cyclic freezing and defrosting. The properties of recycled concrete aggregate significantly influence the quality of concrete. The quality of concrete from RCA is dependent on the initial strength of the construction wastes as well as on the content of mortar that is left on the aggregate and graining [8,9]. The use of concrete aggregate is also reflected in standards and procedures such as [10,11]. The standard [12] makes it possible to use a recycled aggregate in concrete on the condition that it satisfies the criteria concerning the content of possible pollution, which can have an impact on concrete quality and, at the same time, the standard recommends conducting research on the aggregates within the scope of requirements relating to further concrete application. Despite the fact that a series of studies has been performed confirming that recycled concrete aggregates can be an alternative to natural ones, it is still used with great caution. Concretes of average strength are obtained from these aggregates. It requires also applying greater amount of cement in reference to concrete of the same class, obtained from natural aggregates. In order to improve its quality, all the pollutants shall be removed, introducing selective demolition of construction facilities [13]. These aggregates are characterized by great water absorption, which is caused by the mortar adhering to the initial aggregates [9,14]. It should be emphasized that while fulfilling several conditions, it is possible to obtain the concrete from RCA aggregate, which is not different from concrete of similar content on the basis of natural aggregates.

A commonly known research direction, constantly being developed, is the use of ceramic materials as recycled aggregates to concretes. Both red ceramics in the form of bricks, hollow bricks, or tiles as well as white ones (table, sanitary, electric insulators) belong to the materials not subjected to biodegradation, and their utilization is a severe issue [15]. The results of studies on red ceramics as a recycled aggregate are ambiguous. The studies conducted by [16] demonstrated that fragmented bricks can be applied as coarse aggregates to concrete and it does not cause the subsequent deterioration of its strength. However, this type of aggregate is not recommended in reinforced concrete constructions. In the research conducted by [17], two concrete mixes were prepared in the proportion of 1:2:4 (cement:sand:coarse aggregate). One of them on the basis of gravel, the other on crushed bricks. The use of brick aggregates decreased concrete strength up to 80% in relation to samples made from gravel. Moreover, in the research carried out by [18,19,20], it is demonstrated that the total replacement of natural aggregate by red ceramics is virtually impossible due to the significant drop in strength of such a concrete. The attempts have been also made to use white and red ceramics in the form of ground powder with a 0–3 mm grain, as an additive replacing cement to prepare mortars. The research results show that the wastes from usable ceramics from the production of pots and covers can be used in the manufacture of cement mortar. The replacement of both cement and natural aggregate in 15% by wastes from the production of usable ceramics in the cement mortar does not negatively influence its compression strength after 28 days. Nevertheless, the compression strength of examined cement mortars with the addition of ceramics wastes after 150 cycles of freezing and defrosting was subjected to decrease [21,22]. More beneficial results were definitely obtained by the researchers using white ceramics. The studies conducted by [23,24] demonstrated that the concrete made completely from the aggregate of white sanitary ceramics does not differ from natural aggregates in terms of physical and mechanical properties. In the research conducted by [25,26] coarse aggregates were replaced by fragmented ceramic cullets in the amount of 15–20%. Prepared concretes were characterized by similar properties to the ones on the basis of natural coarse aggregates. It was also stated that along with the increase of ceramic matter, the compression strength of concrete also increases. The research conducted by [27,28,29] showed the positive impact of aggregates from ceramic cullets as an additive to concrete, which benefited from its strength and durability and other features as well. Performing their own studies [30,31,32,33] enabled the researchers to demonstrate that the concrete, on the basis of the ceramic cullet aggregates, is resistant to fire temperature and is characterized by great resistance to an aggressive chemical environment. It is possible to obtain mixes of strength parameters that exceed natural aggregate concretes.

## 2. Materials and Methods 

### 2.1. The Description of Concrete Samples Content and Research

#### 2.1.1. Ceramic Cullet

Sanitary ceramic wastes were used in this study and were obtained from used goods produced by one of the Polish sanitary fittings plants (Reybud, Rejowiec Fabryczny, Poland). Ceramics were fragmented into two fractions 0–4 mm and 4–8 mm and subjected to sieve analysis according to [34]. It allowed obtaining the following results of particular fractions (related to aggregate occurring in the designed concrete mix content): (0–0.125 mm) 1.14%, (0.125–0.25mm) 2.79%, (0.25–0.5 mm) 6.29%, (0.5–1.0 mm) 11.21%, (1.0–2.0 mm) 21.79%, (2.0–4.0 mm) 28.07% and (4.0–8.0 mm) 28.57%. Within the frame of conducted research on the aggregate, its crushing strength was measured on the basis of [35].

To determine the mineral content of the ceramic aggregates we used calcium aluminate cement and observed the phase changes taking place during the annealing process of the designed concrete. X-ray diffraction (XRD, Panalytical, Almelo, The Netherlands) was applied. The analysis was conducted by the powder method using X-ray diffractometer Panalytical X’pertPRO MPD (Panalytical, Almelo, The Netherlands) with goniometer PW 3050/60 (Panalytical, Almelo, The Netherlands). As the source of X-radiation, a copper lamp Cu (CuK_α_ = 0.154178 nm) was used. Software X’Pert (Panalytical, Almelo, The Netherlands) Highscore was used for diffraction data processing. The identification of mineral phases was based on data basis PDF-2 release 2010 formalized by JCPDS/ICDD (Panalytical, Almelo, The Netherlands). The microstructure of the examined materials was determined by the Scanning Electron Microscope (SEM) Quanta 250 FEG by FEI (Hilsboro, OR, USA), equipped with the system of chemical content analysis based on the energy dispersion of the X-ray using Energy Dispersive X-ray Spectroscopy (EDS, Panalytical, Almelo, The Netherlands) by EDAX.

#### 2.1.2. Characteristics of Concrete Mix and Samples

Two sample series, which differed solely by the binder, were prepared with the use of an iterative designing method, limiting the substrates of mix commonly used in cements and the appropriate amount of aerating additive. The first series was based on calcium aluminate cement ‘Górkal 70’, and the other comparative one on Portland cement 32.5 R. Only the aggregates from sanitary ceramic wastes were used in each series, fragmented to the two above mentioned fractions. Both series of samples were divided into three series: basic ones and the ones with aerating additive. 0, 5, and 10% degrees of aeration of the concrete mix on the basis of the calcium aluminate and Portland cement were assumed in the research according to [36]. The amounts of aerating mix and the content of particular groups of samples are listed in Table 1. The additive is based on abietic salt, which generates small air bubbles of a diameter from 10 to 300 μm in a concrete mix. It is characterized by a density of 1015 ± 20 kg/m^3^, pH within the range of 10–12, chloride content ≤0.1%, and alkalis ≤7%. Thus far, the aerating mix was used in concretes in order to increase its resilience to low temperatures. The research assumed that the active pores created as a result of aerating would limit the propagation of ruptures, thus increasing the durability of the concrete. Samples were made in the cylindrical shape of diameter 100 mm and the height of 200 mm. In total, there were 90 items, 15 of every described type. Moreover, in order to perform abrasion tests, three tests from each designing group were conducted in the form of cubes 100 × 100 × 100 mm. In compliance with the requirements of the norm, the created samples were demolded after 2 days of cementing, and then they matured for 14 days in a water-bath. The samples then matured in laboratory conditions at a temperature of 20 °C ± 2 °C, and in air conditions with a mean relative humidity of 50% for a further 28 days. Next, in accordance to the PN-EN 1363-1:2012 [37], Fire resistance tests, Part 1: General requirements standard; the samples were kept in the described conditions for 3 months, before thermal stress tests were conducted.

#### 2.1.3. Thermal Processing of Samples

Before compression tests, five samples of each group were annealed to 400 and 800 °C. The remaining samples from each group were not subjected to thermal processing. Samples were annealed in the electric chamber furnace (Thermolab, Warszawa, Poland) PK 1100/5 type. The furnace enables random determining that increase temperature during the course of testing, monitoring, and registration of temperature in real time from 16 measuring thermoelements. The furnace structure is made from square tubes and a stainless steel plate. Heating elements of furnace are made from a resistance wire Kanthal A1 in the spiral shape. 

**Figure 1:**
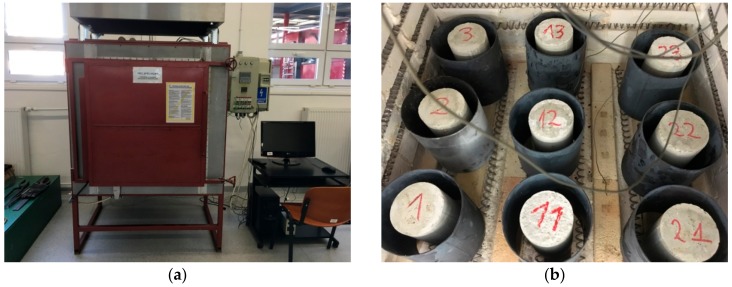
(**a**) Furnace for samples annealing, (**b**) Samples in the furnace chamber secured by steel covers.

The furnace for annealing samples is shown in Figure 1a. In the ceiling there are two chimneys to channel the vapor or to introduce additional measuring sensors. During the research, the temperature in the furnace chamber and the temperature reached on the external surface of selected samples was measured. In order to avoid the damage of furnace chamber, concrete samples were placed in steel covers Figure 1b.

In the course of thermal action, the distribution of temperature was as close as possible to the conditions of a real fire occurring in closed facilities. For this reason, standard distribution temperature-time was assumed as the basis according to [37]. A standard curve temperature-time is described by the formula (1).

T = 20 + 345log (8t + 1),(1)

T—temperature (°C)
t—time (min)

**Figure 2:**
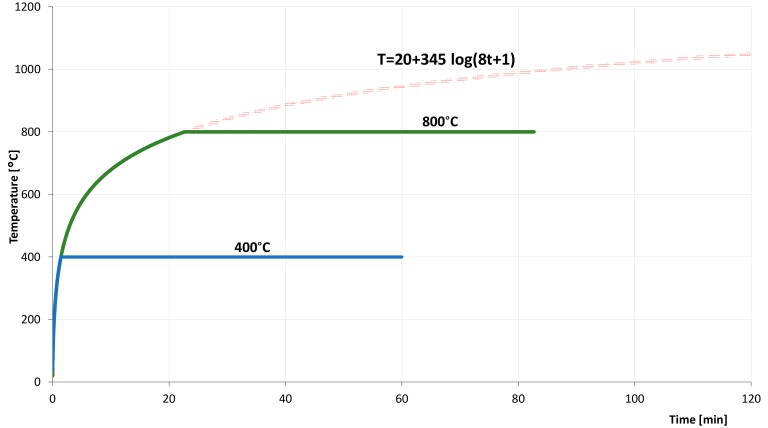
The diagram of temperature distribution while annealing concrete samples.

The increase of thermal loading was implemented in line with the standard curve ‘temperature-time’. After reaching the assumed temperature, according to the research plan amounting to 400 or 800 °C, samples were still annealed for 60 min, which aimed at equalizing the temperature in the whole density of samples. The curve which describes thermal loading of concrete samples is shown in Figure 2. The cooling of the samples was conducted naturally in the chamber of the furnace until they reached room temperature. In the case of samples heated to 800 °C, they were kept in the furnace for the next 36 h after annealing.

Marking of particular samples used to research is presented in Table 2. 

#### 2.1.4. The Research on Abrasion and Compression Strength

The research on concrete abrasion on the basis of sanitary ceramic wastes was performed according to the standard [38]. A test stand was equipped with a Boehme shield along with the equipment. The corundum powder was used as an abrasive according to standard recommendations. Cubic samples had the side length (71.0 ± 1.5) mm. For this purpose, cubes of the side length 100 mm were cut by a stationary saw with a blade directed to cut concrete. The samples were weighed and measured before testing. The sample was mounted in a handle and axially loaded by the strength (294 ± 3) N. The sample was subjected to abrasion in the cycles by the blade rotation in line with standard requirements. In the course of studies, it was monitored whether the corundum powder was evenly distributed on the whole abrasion surface. The blade and sample surface were cleaned, it was rotated by 90° and new abrasive powder was spread after every cycle. In total, 18 samples, 3 per each prepared mix, were subjected to research. They were then measured and weighed in the same way as it was performed during the preparation phase. The research on compression strength of all cylindrical samples was conducted according to [39,40] using the system to examine concrete and mortars: Advantest 9 by Controls (250 kN, Milan, Italy). The system cooperates with a strain gauge bridge with the possibility of distortion readings in four measuring points. The machine is equipped with three frames. The compression frame was used during strength tests within the range of 3000 kN. 

## 3. Results

### 3.1. Characteristics of Ceramic Aggregates

The research was conducted on aggregates of 4–8 mm fraction. The average rate of crushing is 6.75%, which means that the aggregates from sanitary ceramic wastes are resistant to crushing. For comparison, the crushing rate of limestone aggregate is 18–20%, granite about 18%, quartzitic sandstone about 15%; whereas gravel is 12–16%. More importantly, ceramic aggregates were submitted to elementary analysis by means of EDS (Energy Dispersive Spectroscopy) during SEM observations. In order to compare, the analysis of several other aggregates that are frequently used have been also performed. The obtained results are listed in Table 3.

**Figure 3:**
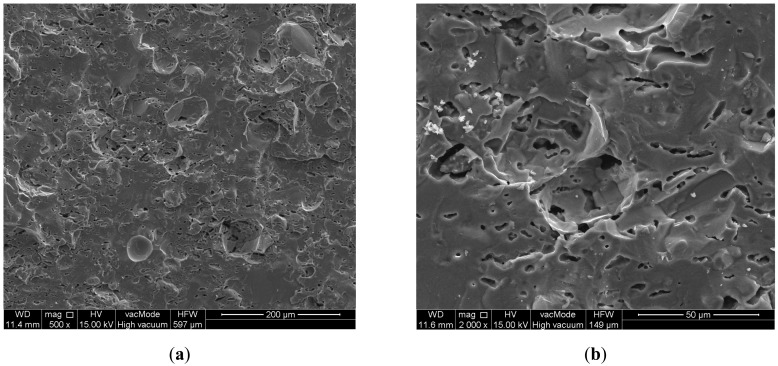
Micrographs of the aggregate ((**a**) magnification 500×, (**b**) magnification 2000×) used in research scanning electron microscope (SEM).

Example photographs made with the SEM scanning microscope depicting the microstructure of the aggregate used in research are presented in Figure 3.

**Figure 4:**
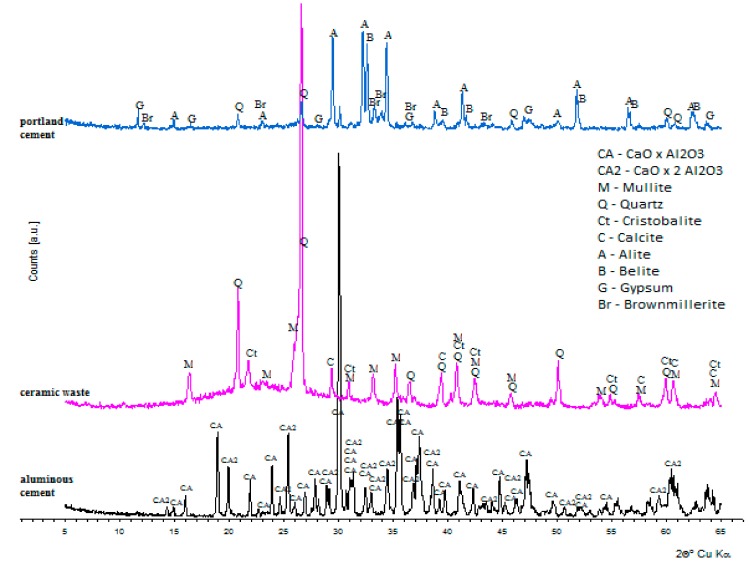
Diffractograms of mineral content of ceramic aggregate.

The minerals of ceramic aggregates used in the designed concrete mixes were also studied. The diffractogram of the aggregates is shown in Figure 4. The main mineral content of the ceramic aggregate constitutes multi-recognized characteristic interplanar distances: d_hkl_ = 0.5376; 0.3425; 0.3390; 02882; 0.2427; 0.2294; and 0.2208 nm. In addition, quartz occurs collaterally, recognized by d_hkl_ = 0.4255; 0.3344, 0.2456; 0.2283; 0.2237; 0.2128; and 0.1981 nm; and cristobalite d_hkl_ = 0.4055; 0.3140; 0.2847; and 0.2486 nm; and calcite d_hkl_ = 0.3861; 0.3040; 0.2283; and 0.2096 nm. Together with the crystal phases in the used aggregates, there was also an amorphous substance (aluminosilicate glaze) whose occurrence on diffractograms is visible by the increase of its background in the range of angles from 15 to 35 (2θ).

### 3.2. Calcium Aluminate Cement

Calcium aluminate cement ‘Górkal 70’ and Portland cement CEM II/B-V 32.5 R were used in this research. The minerals of both cements are presented in Figure 4. In the case of calcium aluminate cement, the predominating crystal phase is calcium monoaluminate (CA), which is accompanied by calcium dialuminate CA_2_. The first was recognized by characteristic interplanar distances d_hkl_ = 0.5956; 0.5529; 0.4687; 0.4052; 0.3718; 0.3309; 0.3199; 0.2974; and 0.2911 nm; and the latter d_hkl_ = 0.6185; 0.4448; 0.3506; 0.2850; and 0.2601 nm. The main mineral phases of used Portland cement are as follows: alite (C_3_S), belite (C_2_S, tricalcium aluminate (C_3_A), and brownmillerite (C_4_AF), which is collaterally accompanied by gypsum and quartz. Alite was recognized by the strongest reflections d_hkl_ = 0.2618; 0.2611; 0.2179; and 0.2174 nm; belite d_hkl_ = 0.2793; 0.2786; 0.2747; and 0.2611 nm; and brownmillerite d_hkl_ = 0.7246; 0.3650; 0.2786; 0.2674; and 0.2646 nm. The occurrence of gypsum is confirmed by reflections d_hkl_ = 0.7634; 0.4292; 0.3067; and 0.2874 nm.

### 3.3. Phase Content and Microstructure of Concretes Containing Calcium Aluminate Cement and 10% of Aeration 

To study the differences in the phases and microstructure, only the samples containing calcium aluminate cement were selected with 10% of aeration (the group of samples marked by C10_20, C10_400, C10_800). The choice of these groups depended on the positive results of research referring to the compression strength of the concrete subjected to temperature load as well as the will to learn about the mechanisms that generate such positive impacts. The studies on mineral identification were conducted as the first regarding the ceramic aggregate itself and unheated calcium aluminate cement; subsequently, the research on concrete samples was performed taking into account basic samples and the ones subjected to temperature loads of 400 and 800 °C. 

**Figure 5:**
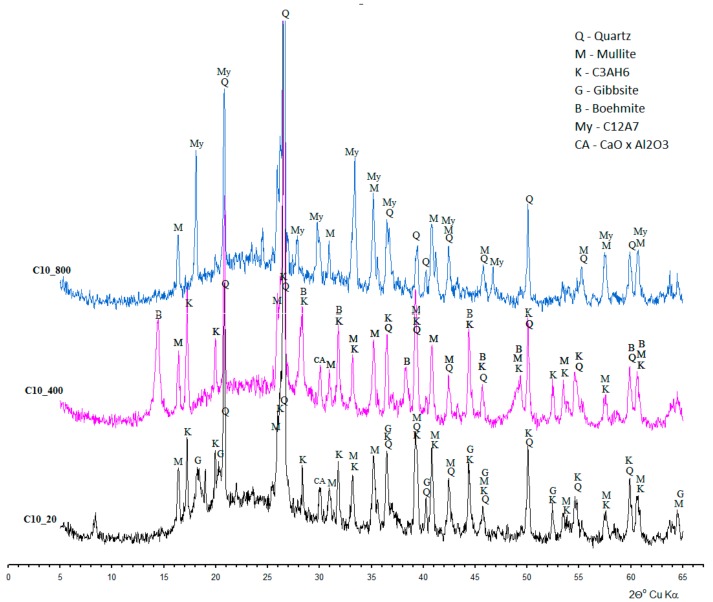
Diffractograms of mineral content of examined concretes containing calcium aluminate cement.

The phases of concretes, dependent on the used cement, are shown in Figure 5. In the minerals of concretes based on calcium aluminate cement, apart from contents from the aggregate and relics of the cement phases (CA and CA_2_), there are new crystalline phases visible on the diffractograms, which are the result of the hydration process and the action of ‘annealing-fire’ temperatures. In the samples of concrete cured at 20 °C (C10_20), as a result of the hydration process, hydrates appear whose chemical content is similar to C_3_AH_6_ (katoite) and AH_3_ (gibbsite) appear. Katoite was recognized by the strongest reflections d_hkl_ = 0.5139; 0.3347; 0.3145; 0.2810; 0.2462; 0.2296; and 0.2040 nm; whereas gibbsite resulted in d_hkl_ = 0.3185; 0.3106; 0.2453; 0.2451; 0.2165; and 0.2049 nm.

The annealing process at the temperature of 400 °C (C10_400) causes the increase of phase content, C_3_AH_6_ type, whereas gibbsite turns into boehmite recognized by the most intensive reflections d_hkl_ = 0.6098; 0.3165; and 0.2347 nm.

**Figure 6:**
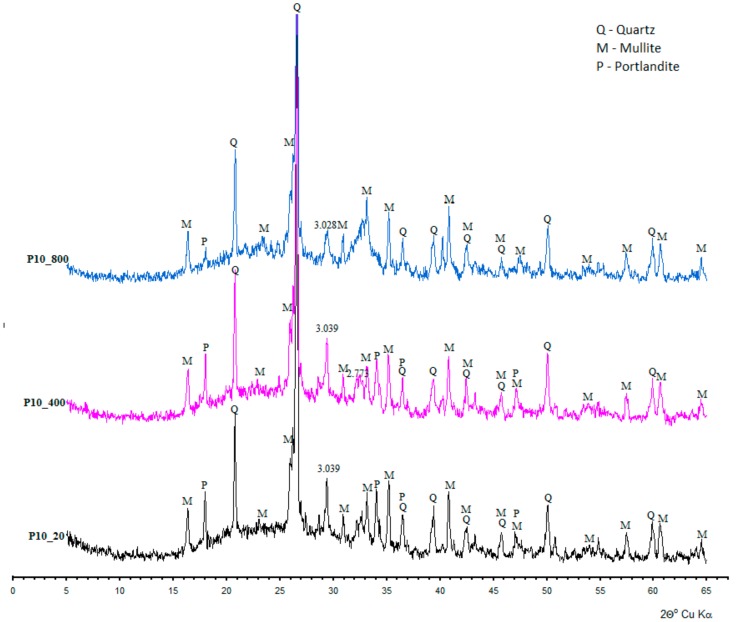
Diffractogram of mineral content of studied concretes containing Portland cement.

In the diffractograms of the sample subjected to temperature action of 800 °C (C10_800), katoite, occurring at lower temperatures, turns into C_12_A_7_ (mayenite) recognized by d_hkl_ = 0.4899; 0.4266; 0.3202; 0.2998; and 0.2681 nm. Concretes, on the basis of Portland cement, demonstrate definitely lower variability in the phase content (Figure 6). Apart from amorphous or poorly ordered hydrated calcium silicate (C-S-H), portlandite predominates and is recognized by characteristic interplanar distances d_hkl_ = 0.4901; 0.2628; and 0.1927 nm, and whose quantity decreases along with the increase of annealing temperature.

**Figure 7:**
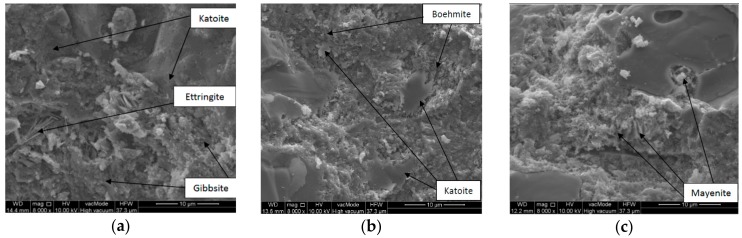
SEM micrograph of sample: (**a**) C10_20, (**b**) C10_400, (**c**) C10_800.

The microstructural observations of the designed concretes are shown in Figure 7. The observations with the use of SEM demonstrated that the ceramic aggregate and calcium aluminate cement have an impact on the change in concrete microstructure in the condition of high temperature. In the SEM micrographs of concretes C10_20 there are a distinct share of irregularly occurring pores, in which there are relics of monocalcium aluminate (CA). Mineral phases predominating in this concrete are as follows; katoite formulates very fine crystalline toppings, and gibbsite, shaped in the form of bars and posts, with accumulations that form dripstone concentrations (Figure 7a). The microstructure of the concrete samples C10_400 is basically shaped by morphological relations between boehmite, which was formed as a result of the thermal processing of gibbsite, and irregularly shaped katoite panels (Figure 7b). In relation to concrete C10_20. There is the almost complete lack of pores of large dimensions >10 µm. Porosity is solely related to the presence of empty spaces between dripstone boehmite forms. The impact of the annealing temperature on the concrete structure is distinctly visible in the sample C10_800 (Figure 7c). Along with phase changes related to the transformation of katoite into mayenite, there are some fractures and microcracks in the contact zone: aggregate-cement paste.

**Figure 8:**
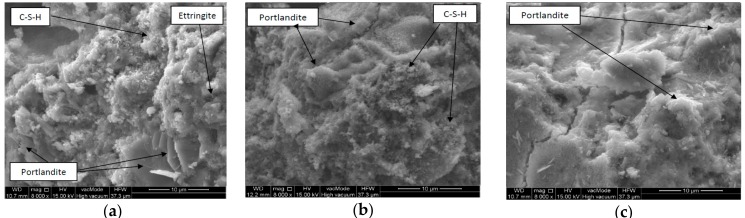
SEM micrograph of sample: (**a**) P10_20, (**b**) P10_400, (**c**) P10_800.

In the concretes based on Portland cement, the SEM micrograph shows that there is a fine-grained and locally fine-fibrous ettringite phase whose crystal concentration reaches the dimension about 10 µm. There is a distinct presence of portlandite occurring in the form of relatively large bars of hexagonal outline and layered structure. In the SEM microphotographs of the concretes, needle forms of ettringite can be observed whose length amounts to 0.5 µm (Figure 8a). The impact of the annealing process of concretes with the use of Portland cement as a binder on the microstructure is visible by the concentrations blur of the dehydrated calcium aluminate C-S-H type, and the portlandite crystals as well as the disappearance of ettringite (Figure 8b). The SEM observations also demonstrate the increase of porosity and the presence of numerous fractures (Figure 8c).

### 3.4. The Values of Compression Strength and Abrasion Level of Concrete Samples

**Figure 9:**
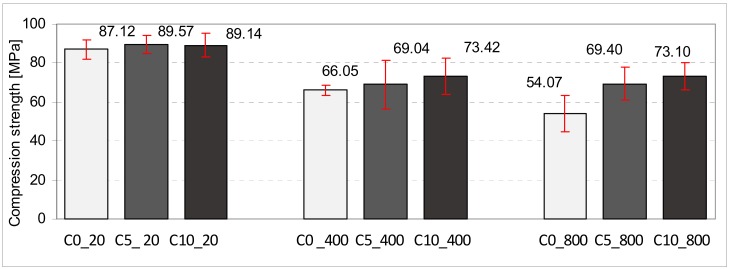
The average values with standard deviations of compression strength of cubic samples containing calcium aluminate cement.

**Figure 10:**
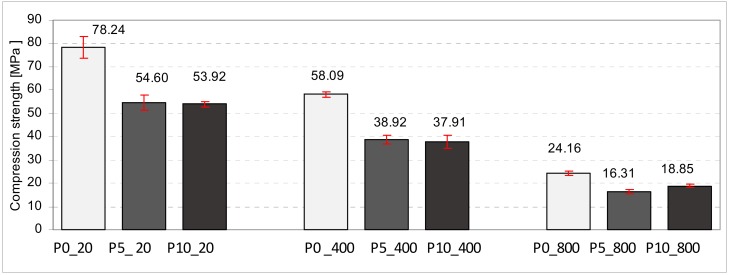
The average values with standard deviations of compression strength of cubic samples containing Portland cement.

**Figure 11:**
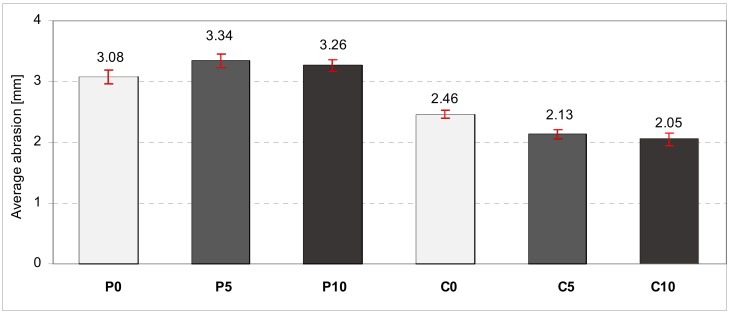
The average values with standard deviations referring to the abrasion of concrete samples.

The diagrams presented below show the average values together with standard deviations of the compression strength of cubic samples (Figure 9 and Figure 10) and abrasion determined on the basis of height loss (Figure 11). Figure 10 shows the results referring to the series of samples containing Portland cement, whereas Figure 9 illustrates the results of samples containing calcium aluminate cement. Particular series were marked in the graphs according to Table 2. Basic samples, which were not subjected to annealing, are marked in the graphs by a white color. Samples annealed at the temperature of 400 °C are marked in grey and samples annealed at 800 °C are marked in black.

The series of examined samples with Portland cement contained 45 items. It shall be emphasized that while annealing at high temperatures, none of the samples were damaged. In the group of basic samples, the greatest compression strength was demonstrated by the samples without the aerating additive. Their average compression strength was greater by about 32% in comparison to the samples that contained aeration of 5 and 10%. Annealing had an adverse effect on samples’ strength on the basis of the Portland cement. The higher the annealing temperature, the lower the compression strength of the samples. In the group of samples subjected to annealing at 400 °C, the average compression strength of the base samples (without the aerating additive) dropped by about 27% in comparison to samples that were not subjected to annealing. Moreover, in reference to samples containing 5 and 10% degrees of aeration, their compression strength decreased within the range of 28–10% in relation to samples that were not subjected to thermal processing. After the analysis of samples subjected to initial thermal processing at 800 °C, it shall be stated that their compression strength is the lowest for every tested group of samples. The average strength of base samples without the aerating additive is lower by about 71% in reference to samples that were not subjected to thermal processing. Nevertheless, their compression strength is the greatest in comparison to aerated samples by 5 and 10%. The lowest average strength was obtained by samples containing a 5% degree of aeration, which were simultaneously subjected to thermal processing in 800 °C.

In the case of samples made from calcium aluminate cement, 45 samples were examined in total. In this case, however, the average compression strength of the base samples containing 0, 5, and 10% degrees of aeration was nearly identical. The difference in the average strength was only about 2 MPa. The higher the annealing temperature, the lower the average compression strength of particular groups of samples. In the case of samples annealed at 400 °C, the lowest average strength was observed for samples that were not subjected to aeration. Their strength dropped by about 26% in comparison to samples that were not subjected to thermal processing. In the case of samples subjected to aeration, the drop was considerably lower. In relation to samples with 5% aeration it was 22%, whereas for samples containing 10%, the degree of aeration was 18%, comparing the same groups of samples that were not annealed.

In the case of samples that were initially annealed at 800 °C, the greatest drop in the average strength was demonstrated by the samples without an aerating additive. It was 38% in comparison to samples that were not subjected to thermal processing. In the case of samples with the aerating additive of 5% and 10%, the decrease in strength was nearly identical as in the case of samples annealed to the temperature of 400 °C.

It must be highlighted that in the case of concrete made from calcium aluminate cement, the drop in concrete strength after initial annealing at 400 and 800 °C is considerably lower than in the case of samples made from Portland cement.

Our study conducted on abrasion with the use of a Boehm blade was aimed at determining the height loss susceptibility of particular samples. The research was performed on base samples that were not subjected to annealing. The samples made from all designed concrete mixes were tested. The greatest level of abrasion was observed in the groups of samples C0_20 and P0_20. The aeration led to the decrease of abrasion level by about 10% in the case of samples containing calcium aluminate cement. The lowest level of abrasion was observed in the samples with Portland cement and 5% of aeration, however, these differences were not substantial in comparison to samples containing Portland cement. When comparing the obtained results with those of [31], it should be noted that the results of the durability tests in normal temperature were similar. However, the results of tests conducted after thermal load differed significantly. It is caused by the fact that in the research paper [31], thermal load was applied in a different method, which was modeled after tests for heat resistant cement, contrasting to those described in this article, where the temperature-time norm curve was used.

## 4. Conclusions

The obtained results showed that there is the likelihood of waste matter use in the form of sanitary ceramic cullet as the aggregate to concrete of high resistance to fire conditions. The type of cement applied to their preparation has a significant impact on the microstructure of concretes subjected to annealing. Concretes that contained calcium aluminate cement are characterized by the variability of mineral content, which is the effect of progressing the hydroxylation process of the cement phase surfaces accompanied by the release of calcium and aluminum ions. After exceeding the solubility equilibrium of these compounds, new phases start to crystalize such as, katoite and mayenite, whereas aluminum hydroxides are transformed from gel forms into more and more ordered forms such as gibbsite and boehmite. The formation of new phases decreases considerably the amount of pores in the slurry. Nevertheless, in the case of concretes with Portland cement, the annealing process causes dehydration and dehydroxilation of hydrated phases that liberate water vapor in the pores and generate pressure, leading to matrix cracking, which directly decreases the mechanical properties of these concretes.

It was demonstrated that by using the binder in the form of calcium aluminate cement, the obtained concrete demonstrates high resistance to compression. The impact of the aerating additive showed a slight increase of strength in the group of base samples containing calcium aluminate cement. The application of the aerating additive for the group of samples subjected to annealing in the temperature of 400 and 800 °C reduced the decrease of strength. The results at the temperature of 800 °C are particularly significant since in the case of fire developed in closed rooms, it corresponds to the temperature value occurring in the fire developed under the ceiling. It shall be stated that the use of additive led to the almost identical decrease of strength at 400 and 800 °C. The situation is completely different in the case of concrete made from Portland cement. The greatest average compression strength was demonstrated by the base samples (without aerating additive), which were not subjected to annealing. The application of the aerating additive both at 5% and 10% to this concrete resulted in a greater drop in strength than for the concrete without the additive both at room temperature and after annealing. It shall be noticed that in the case of concrete on the basis of calcium aluminate cement and Portland cement, there were no thermal phenomena and explosive chipping of concrete while annealing, as it took place in the case of samples in the shape of cubes. Moreover, it shall be stated that samples used in this research were stored in a dry room at the temperature of 20 °C through the period of 12 months, which is most likely the reason why the described phenomena did not appear. Furthermore, the research on abrasion demonstrated that the use of aerating additive causes the decrease of abrasion with calcium aluminate and Portland cement. The application of additive to concrete based on Portland cement resulted in the decrease of the abrasion level of samples. Demonstrated studies confirmed the possibility of a ceramic cullet application as the aggregate to concrete. Such an approach contributes to the reduction of natural resource extraction and use, and enables the utilization of wastes of this type.

## Figures and Tables

**Table 1:** The comparison of prepared concrete mixes in 1 m^3^ of concrete mix.

Group	Group I			Group II		
Marked samples	P0	P5	P10	C0	C5	C10
Calcium aluminate cement “Gorkal 70” (kg)	488	488	488	-	-	-
Portland cement 32.5 R (kg)	-	-	-	488	488	488
Aggregate 0–4 mm (kg)	997.14	997.14	997.14	997.14	997.14	997.14
Aggregate 4–8 mm (kg)	398.86	398.86	398.86	398.86	398.86	398.86
Water (L)	199	199	199	199	199	199
Aerating additive (kg)	-	0.244	0.488	-	0.488	0.976

**Table 2:** Marking of samples used to research.

Group	Portland Cement			Calcium Aluminate Cement		
Degree of concrete aeration	0%	5%	10%	0%	5%	10%
Basic samples not subjected to annealing 20 °C	P0_20	P5_20	P10_20	C0_20	C5_20	C10_20
Samples annealed in 400 °C	P0_400	P5_400	P10_400	C0_400	C5_400	C10_400
Samples annealed in 800 °C	P0_800	P5_800	P10_800	C0_800	C5_800	C10_800

**Table 3:** The comparison of chemical content of selected concrete aggregates.

Type of Aggregate	Compounds Content (%)								
	SiO_2_	Al_2_O_3_	Fe_2_O_3_	CaO	MgO	Na_2_O	K_2_O	TiO_2_	Other
Gravel	16.7	8.0	1.0	31.8	40.8	0.9	0.8	-	-
Granite	74.8	14.2	-	1.6	0.6	5.6	2.8	-	0.4
Red clay ceramics	51.7	18.2	6.1	6.1	2.4	0.2	4.6	0.8	10.0
Sanitary ceramics	67.6	24.1	0.6	-	0.4	1.3	3.0	-	3.0
